# Effects of social anxiety and self‐schemas on the impact and meaningfulness of positive versus negative social autobiographical memories

**DOI:** 10.1111/bjc.12504

**Published:** 2024-09-16

**Authors:** Katie E. Martin, Sophie M. Kudryk, David A. Moscovitch

**Affiliations:** ^1^ Department of Psychology and Centre for Mental Health Research and Treatment University of Waterloo Waterloo Ontario Canada

**Keywords:** appraisals, autobiographical memory, impact, self, self‐schemas, social anxiety

## Abstract

**Objective:**

Social anxiety is characterized by maladaptive self‐schemas about being socially undesirable. Self‐schemas are deeply held beliefs which are derived from negative autobiographical memories of painful social experiences. In contrast to the plethora of past research on negative memories in social anxiety, almost no research has investigated objectively *positive* social autobiographical memories. In this preregistered study, we examined the effects of social anxiety and self‐schemas on the appraised impact and meaningfulness of retrieved positive versus negative social autobiographical memories.

**Method:**

Participants recruited via Prolific (final *n* = 343) were randomized to one of two conditions in which they were instructed to retrieve, orally narrate, and appraise a positive or negative social autobiographical memory of a specific experience from their personal past where they felt either valued or unvalued, respectively.

**Results:**

Results demonstrated that participants rated their positive memories as more impactful and meaningful than negative memories overall, but this effect was reversed for participants who endorsed having either stronger negative self‐schemas or greater social anxiety symptoms, for whom negative memories were more impactful. Additionally, participants who endorsed having stronger positive self‐schemas rated their negative memories as significantly less impactful and their positive memories as nearly more impactful.

**Conclusion:**

Together, these results elucidate how self‐schemas and social anxiety are related to autobiographical memory appraisals, paving the way for future research on memory‐based therapeutic interventions for social anxiety disorder.

**Practitioner Points**

- This study highlights how social anxiety symptoms and positive and negative self‐schemas are linked to appraisals of impact and meaningfulness of positive versus negative social autobiographical memories in a community sample.
- Results imply that interventions designed to weaken negative self‐schemas and bolster positive self‐schemas may help reduce the maladaptive influence of negative social memories and amplify the adaptive effects of positive social memories.
- Results have the potential to guide future research on memory‐based therapeutic interventions for social anxiety disorder.

## INTRODUCTION

Social anxiety disorder (SAD) is characterized by anxious preoccupation with the potential threat of social scrutiny and negative evaluation (American Psychiatric Association, 2013; Stein & Stein, 2008). SAD represents the extreme high end of the social anxiety continuum, the point after which social anxiety symptoms begin to cause significant distress or functional impairment (Ruscio, 2010). People who struggle with social anxiety, including those with SAD, tend to view themselves as being socially undesirable (Clark & Wells, 1995; Hofmann, 2007; Moscovitch, 2009; Rapee & Heimberg, 1997). This negative perception of self is encapsulated within negative mental self‐images that are derived from past distressing social autobiographical experiences and reflect how they believe they appear to others in social situations (Hackmann et al., 1998, 2000).

The autobiographical memory system is comprised of a specialized neural network that encodes personal experiences and is responsible for constructing a sense of self that is stable and continuous across time (Conway & Pleydell‐Pearce, 2000; Çili & Stopa, 2015; Moscovitch, Moscovitch, & Sheldon, 2023). This function of self‐continuity is performed, at least in part, through the influence of self‐schemas, which are knowledge structures about the self that serve as a lens through which new information is processed. Past research has shown that individuals with high social anxiety (HSA) tend to prioritize the processing (i.e., greater attention, perception, and encoding) of negative information and events that align with their social anxiety (Hirsch & Clark, 2004; Morgan, 2010; Peschard & Philippot, 2016). For example, aversive social memories are remembered more easily and described in greater detail than non‐aversive memories for those with social anxiety (Moscovitch et al., 2018). Furthermore, in a series of studies, Krans et al. (2014, 2017) found that autobiographical self‐defining memories recalled by those with HSA or SAD were more negative and contained more themes related to social anxiety. Hertel et al. (2008) similarly found that participants with social anxiety generated endings to ambiguous social scenarios that were more negative and associated with socially anxious themes compared to control participants. Later, recollections of these scenarios aligned with their self‐generated endings, substantiating the biasing effects of social anxiety‐related schemas on memory encoding for social information. Thus, the relationship between schemas and memories is bidirectional and reciprocal, such that schemas shape the encoding and consolidation of social experiences (Conway, 2005; Romano, Ma, et al., 2020) and are, in turn, shaped by experiences that reinforce existing perceptions of self (Moscovitch, Moscovitch, & Sheldon, 2023).

In contrast to the plethora of studies on negative autobiographical memories and social anxiety, research on positive memories and positive self‐schemas is relatively limited. There is evidence that socially anxious individuals tend to experience positivity deficits (Kashdan et al., 2011), where positive social events are perceived more negatively (Alden et al., 2008), positive social memories are less accessible (Moscovitch et al., 2011), and positive information is less readily incorporated into self‐views (Koban et al., 2017). However, no prior studies have investigated the effects of social anxiety and self‐schemas on the retrieval and appraisal of positive social autobiographical memories and how these effects might compare to those of negative social autobiographical memories. This study was designed to fill these gaps in the literature.

How might social anxiety and self‐schemas be linked to appraisals of positive versus negative autobiographical memories? As noted above, past research has demonstrated that individuals with SAD often interpret socially adverse memories more negatively than those without the disorder; these memories deeply influence self‐perception, intensifying negative affect during recall (Moscovitch et al., 2018). Importantly, while the factual details of an event remain constant, the attributed meaning can evolve over time. Investigating the meaningfulness and impact of positive versus negative social autobiographical memories can shed light on the memories that individuals consider central to their identity and their perceptions of self, others, and the world. Additionally, by understanding which memories hold personal significance in terms of their meaningfulness and impact, we may gain insights that help clinicians guide socially anxious individuals in reshaping their memory appraisals or self‐schemas through the activation of specific memories.

In this study, participants were randomly assigned to retrieve and appraise either a positive or negative social autobiographical memory. We hypothesized that the effects of memory valence (i.e., positive vs. negative memory condition) on rated impact and meaningfulness would be moderated by the strength of participants' social anxiety symptoms as well as their negative and positive self‐schemas. Specifically, we expected that greater social anxiety or more negative self‐schemas would amplify impact and meaningfulness in the negative memory condition but would constrain impact and meaningfulness in the positive memory condition. We also expected that positive self‐schemas would amplify impact and meaningfulness in the positive condition but constrain impact and meaningfulness in the negative memory condition.

## METHOD

### Transparency and openness

#### Preregistration

Hypotheses, method and the data analytic plan were preregistered on the Open Science Framework (OSF) website at https://osf.io/g52pe.

#### Data, materials, code and online resources

The final de‐identified data files and syntax are publicly available at the OSF website https://osf.io/yrz98.

#### Reporting

We report how we determined our sample size, all data exclusions, all manipulations and all measures used in this study.

#### Ethical approval

This research was conducted with approval from the authors' institution Human Research Ethics Committee. Participants provided their written informed consent to participate.

### Participants

A total of 400 participants were recruited from Prolific, an online crowdsourcing website, in February 2023. Given the established effects of ageing on autobiographical memory and consistent with prior research on autobiographical memory recall (Levine et al., 2002), eligible participants were required to be between the ages of 18 and 35 years. Participants were excluded if they were outside this age range, if they reported a lack of fluency in the English language (Hong et al., 2022), endorsed having no audio functionality on their computer which would have prevented them from hearing the audio instructions associated with the study or indicated they did not have a microphone connected to their computer in order to record verbal responses within the study. After excluding invalid data (see Supplemental Materials), the final study sample consisted of 343 participants. The study lasted approximately 30 min, and participants received financial remuneration following the completion of the study in accordance with Prolific's fair wage policy. Table 1 summarizes participants' demographic characteristics across conditions and overall. Power analyses are described in Supplemental Materials.

**TABLE 1:** Demographic characteristics of the study sample overall and in each condition.

	Positive condition (*n* = 175)	Negative condition (*n* = 167)	Overall (*N* = 343)
Age in years, M (SD)	27.97 (4.697)	27.65 (4.865)	27.81 (4.775)
Gender (%)			
Male	27.11	27.40	54.52
Female	22.74	19.82	42.56
Non‐binary	0.87	0.58	1.45
Prefer not to respond	0.29	0.87	1.16
Ethnicity (%)			
African	2.33	1.75	4.08
Asian	2.04	3.79	5.83
Black/Afro‐Caribbean/African/African American	3.50	2.33	5.83
East Asian	0.58	0.87	1.45
Hispanic Latino	0.00	0.29	0.29
Indigenous/Métis/Inuit	0.29	0.00	0.29
South Asian/Southeast Asian	3.79	4.66	8.45
White/European	41.11	34.69	75.80
Prefer to self‐identify	0.29	2.04	2.33
Prefer not to respond	0.00	1.46	1.46
Country of residence (%)			
Canada	2.62	2.04	4.66
Ireland	0.00	0.29	0.29
United Kingdom	46.36	46.06	92.42
United States	2.04	0.58	2.77

### Measures

#### Outcome variables

Memory meaningfulness and impact were assessed using subscales from the subjective memory rating items in Moscovitch et al. (2018). The meaningfulness subscale was composed of three items that were rated from 1 (*not at all*) to 10 (e*xtremely*). Items included: ‘The event in my memory was important to me when it occurred’, ‘The event in my memory is important to me now’ and ‘The event in my memory is a central part of my life story’. Items were summed and the Cronbach's αwas .75. The impact subscale was also composed of three items that were rated from 1 (*not at all*) to 10 (*extremely*). Items included ‘This event influenced how I view myself’, ‘This event influenced how I view other people’ and ‘This event influenced how I view the world’. Items were summed and the Cronbach's *α*was .82.

#### Primary predictor variables

##### Memory recall task

Participants in the positive memory condition were asked if they could recall a personal social memory where they felt accepted, connected or valued by others, while participants in the negative memory condition were asked if they could recall a personal social memory where they felt rejected, lonely or unvalued.

##### Trait Social Anxiety

The Social Phobia Inventory (SPIN; Connor et al., 2000) is a 17‐item self‐report scale that assesses the extent to which an individual experienced symptoms of social anxiety during the past week and includes items such as ‘I am bothered by blushing in front of other people’. Participants rated how much the items had affected them in the past week on a scale from 0 (*not at all bothersome*) to 4 (*extremely bothersome*). The SPIN has demonstrated strong psychometric properties in previous studies (Antony et al., 2006; Connor et al., 2000). In this study, Cronbach's αfor the SPIN was .94.

##### Self‐Schemas

The Brief Core Schema Scale (BCSS; Fowler et al., 2006) is a 24‐item questionnaire assessing positive and negative schemas that individuals hold of themselves and others. We administered the entire measure, but analysed only the positive and negative self‐subscales, as outlined in our preregistered plan. Items such as ‘I am interesting’ or ‘I am worthless’ were rated from 1 (*slightly*) to 4 (*totally*), based on how one appraises oneself in that moment. The scale developers reported that it has strong validity and reliability (Fowler et al., 2006). In this study, Cronbach's *α*'s for the BCSS self‐negative and self‐positive subscales were .91 and .90, respectively.

#### Additional measures

Participants estimated the age of their recalled memory using predefined time frames such as ‘this memory was from today or the past week’, with five choices offered reflecting increasingly distant time periods (see Table 2; Söderlund et al., 2014). A brief demographics questionnaire was also administered at the end of the study, along with a brief condition adherence check and a participant attentiveness and engagement measure to assess data validity (see Supplemental Materials for more information). Additional measures specified in the OSF preregistration plan are not included in this paper and will be reported elsewhere.

**TABLE 2:** Descriptive statistics for primary measures overall and across conditions.

Reported measures	Positive condition	Negative condition	Overall
*N*	175	168	343
M_SPIN_ (SD)	27.66 (14.81)	25.36 (16.12)	26.53 (15.48)
M_BCSS (Negative)_ (SD)	4.25 (4.97)	4.36 (5.39)	4.30 (5.17)
M_BCSS (Positive)_ (SD)	12.08 (5.36)	11.88 (5.60)	11.99 (5.47)
M_Impact_ (SD)	17.04 (7.13)	16.07 (6.91)	16.90 (7.15)
M_Meaningfulness_ (SD)	18.42 (6.30)	15.42 (5.56)	18.17 (7.15)
Memory age estimation (%)			
From today or past week	4.37	2.33	6.70
2–6 weeks old	9.04	8.16	17.20
6–18 months old	22.74	17.49	40.23
5–15 years old	13.70	16.03	29.73
15+ years old	0.87	4.37	5.24
Prefer not to answer	0.29	0.58	0.87

*Note*: Impact rating items (range of scores = 3–30); meaningfulness rating items (range of scores = 3–30).

Abbreviations: BCSS, Brief Core Schema Scale (subscales range of scores = 0–24); SPIN, Social Phobia Inventory (range of scores = 0–68).

### Procedure

After providing consent, participants completed the study online through Qualtrics. They were informed that the study would require them to verbally recollect a social memory from their personal past and were instructed to complete the study in a comfortable and private setting that allowed them to speak openly about their personal experiences.

Participants were randomly assigned to one of two memory recall conditions in which they were instructed to retrieve either a positive or negative social autobiographical memory. If participants were unable to retrieve a relevant memory, they received up to three sets of prompts (see Supplemental Materials). Participants who could recall a memory provided a brief 2–3 sentence summary and completed the memory validity check to verify that they retrieved a true autobiographical memory (see Supplemental Materials).

Participants described their memory in as much detail as possible out loud into their computer microphone. Audio recordings of memory recollections were captured and automatically transcribed using the Phonic software program. Then, they rated the age of their memory, along with the memory impact and meaningfulness. Last, participants completed the SPIN, BCSS self‐negative and self‐positive subscales, study engagement and condition adherence measures, and the brief demographic questionnaire.

### Data analytic plan for primary analyses

Data were analysed in IBM SPSS Statistics 23 (2015). As outlined in the preregistered plan, hierarchical linear regression analyses were used to test the main and interactive effects of predictor variables, which consisted of: (a) positive versus negative memory retrieval (memory valence) condition and (b) levels of social anxiety (SA) symptoms (SPIN scores) *or* negative or positive self‐schema strength (BCSS negative or positive self‐schema subscale scores) on the outcome variable, which consisted of either appraised memory impact and meaningfulness in separate analyses. Continuous predictor variables were grand‐mean centred for all regression models. Memory valence condition and either SPIN scores or BCSS scores were entered in the first step, and an interaction term was entered in the second step.

Exploratory analyses were also conducted to examine the effects of the three‐way interaction between memory valence, SA symptoms and self‐schemas in a single model.

## RESULTS

### Data screening

For each self‐report questionnaire and subscale, missing data for individual items were imputed using the expectation–maximization method when a majority of items were present and results of Little's (1988) MCAR tests ascertained that the data were missing completely at random (Osborne, 2013). Non‐MCAR data from the SPIN were not imputed and were excluded from analyses. Outlier analyses detected no significant outliers in the dataset. Results of study engagement and condition adherence measures demonstrated a high level of engagement and adherence for all participants included in analyses (see Table 3).

**TABLE 3:** Descriptive statistics for adherence checks and engagement checks across conditions.

Item numbers	Positive condition	Negative condition
*N*	175	167
Adherence Item 1, *M* (*SD*)	4.30 (0.78)	4.09 (0.88)
Adherence Item 2, *M* (*SD*)	4.10 (0.78)	3.89 (0.85)
Adherence Item 3, *M* (*SD*)	4.07 (0.86)	3.89 (0.84)
Adherence Item 4, *M* (*SD*)	4.06 (0.87)	3.93 (0.93)
Total	16.53 (2.60)	15.80 (2.83)
Engagement Item 1, *M* (*SD*)	4.34 (0.91)	4.26 (0.88)
Engagement Item 2, *M* (*SD*)	4.77 (0.63)	4.62 (0.89)
Engagement Item 3, *M* (*SD*)	4.84 (0.45)	4.77 (0.52)
Total	13.88 (1.67)	13.57 (1.86)

*Note*: Adherence Check items were rated from 1 (*not at all*) to 5 (*extremely*). Engagement Check items were rated from 1 (*not at all*) to 5 (*a great deal*).

### Primary analyses

Tables 1 and 2 show participants' demographic characteristics and descriptive statistics for the primary study measures, both overall and across conditions.

#### Effects of memory valence condition and social anxiety

A two‐step hierarchical regression analysis was used to examine the main effects and two‐way interaction between memory valence (positive vs. negative condition) and SA symptoms (SPIN scores) on memory meaningfulness and impact, with main effects entered on step one and interaction effects entered on step two. Full regression output is presented in Table S2.

##### Meaningfulness

Results indicated a main effect of memory valence on meaningfulness such that participants who retrieved a positive memory tended to rate their memory as more meaningful than those who retrieved a negative memory (*β* = .472, *t* = 9.597, *p* < .001). However, there was no significant main effect of SA symptom severity on meaningfulness (*β* = .054, *t* = 1.091, *p* = .276). Overall, the first step in this model was significant, *F* (2, 322) = 47.981, *p* < .001, with memory valence and SA symptoms accounting for 23.1% of the variance in participants' ratings of memory meaningfulness. Contrary to our hypothesis, there was no significant interaction between memory valence and SA (*β* = −.114, *t* = −1.717, *p* = .087).

##### Impact

**FIGURE 1:**
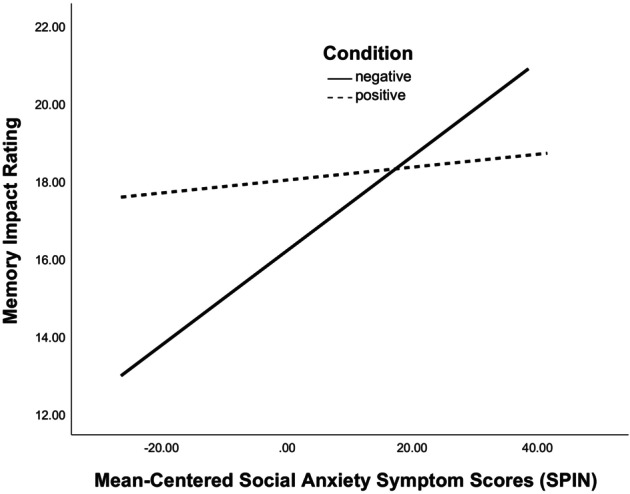
Interaction effect between self‐reported social anxiety symptoms (SPIN) and memory valence (assigned condition) on appraised memory impact. SPIN, Social Phobia Inventory.

Results revealed a significant main effect of memory valence on impact such that participants in the positive memory condition rated their memory as more impactful than those in the negative memory condition (*β* = 1.816, *t* = 2.335, *p* = .020). There was also a significant main effect of SA symptoms on impact (*β* = .122, *t* = 3.535, *p* < .001), such that participants who had higher SA tended to rate their memory as more impactful. Overall, the first step in this model was significant, *F* (2, 323) = 7.357, *p* < .001, with memory valence and SA accounting for a total of 4.4% of the variance in participants' ratings of memory impact. The second step in the model, with the addition of the interaction term, was also significant, *F* (3, 323) = 6.393, *p* < .001, accounting for a total of 5.7% of the variance in participants' ratings of memory impact, Δ*R* ^2^ = .013. Consistent with hypotheses, there was a significant interaction of memory valence and SA symptoms on impact (*β* = −.105, *t* = −2.077, *p* = .039). Follow‐up simple slopes analyses revealed that participants reporting higher SA tended to rate their negative memories as more impactful (*β* = .282, *t* = 3.687, *p* < .001), but SA symptoms were not significantly related to impact ratings of positive memories (*β* = .034, *t* = .432, *p* = .666; see Figure 1).

#### Effects of memory valence and negative self‐schemas

In a second set of planned analyses, we examined the main effects and two‐way interaction of memory valence (positive vs. negative condition) and strength of negative self‐schemas (BCSS negative self‐schema scores) on memory meaningfulness and impact. Regression outputs for all models are presented in Tables S3 and S4.

##### Meaningfulness

Results revealed a main effect of memory valence on meaningfulness such that participants who recalled a positive memory tended to appraise their memory as more meaningful than those who recalled a negative memory (*β* = .474, *t* = 9.812, *p* < .001). Results also showed a main effect of negative self‐schema strength on memory meaningfulness such that individuals with stronger negative self‐schemas tended to appraise their memory as more meaningful (*β* = .110, *t* = 2.269, *p* = .024). Overall, the first step of the model was significant, *F* (2, 330) = 50.527, *p* < .001, with memory valence and negative self‐schema strength accounting for 23.6% of the variance in participants' memory meaningfulness ratings. Contrary to our hypothesis, there was no significant interaction effect of memory valence and negative self‐schema strength on memory meaningfulness (*β* = −.114, *t* = −1.725, *p* = .085).

##### Impact

**FIGURE 2:**
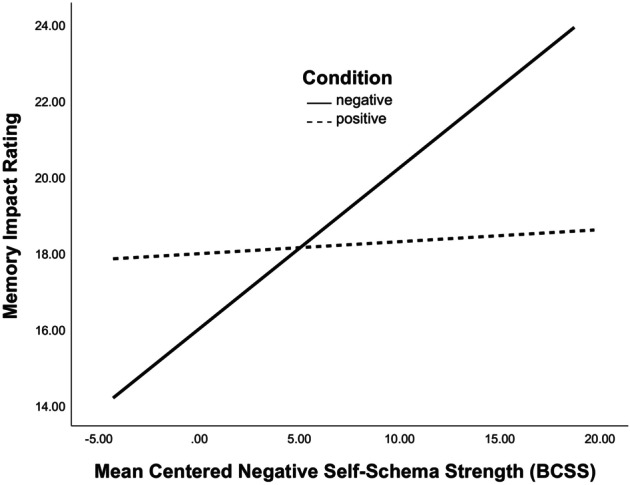
Interaction between negative self‐schema strength ratings (BCSS) and memory valence (assigned condition) on appraised memory impact. BCSS, Brief Core Schema Scale.

Results indicated a main effect of memory valence on impact such that participants who recalled a positive memory tended to appraise their memory as more impactful than those who recalled a negative memory (*β* = 1.967, *t* = 2.588, *p* = .010). Results showed there was also a main effect of negative self‐schema strength on memory impact such that individuals with stronger negative self‐schemas tended to appraise their memory as more impactful (*β* = .423, *t* = 4.207, *p* < .001). Overall, the first step was significant, *F* (2, 331) = 8.385, *p* < .001, with memory valence and negative self‐schema strength accounting for 4.8% of the variance in participants' memory impact ratings. The second step, with the addition of the interaction term, was also significant, *F* (3, 331) = 8.071, *p* < .001, accounting for a total of 6.9% of the variance in participants' ratings of memory impact, Δ*R* ^2^ = .020. In support of hypotheses, the interaction between memory valence and negative self‐schema strength on memory impact was significant (*β* = −.391, *t* = −2.670, *p* = .008). Simple slopes analyses revealed that negative memories were rated as more impactful by participants who endorsed stronger negative self‐schemas (*β* = .331, *t* = 4.443, *p* < .001), but the strength of negative self‐schemas was not associated with impact ratings of positive memories (*β* = .022, *t* = .282, *p* = .778) (see Figure 2).

#### Effects of memory valence and positive self‐schemas

In a third set of planned analyses, we examined the main effects and two‐way interaction of memory valence (positive vs. negative condition) and strength of positive self‐schemas (BCSS positive self‐schema scores) on memory meaningfulness and impact.

##### Meaningfulness

Results revealed a main effect of memory valence on meaningfulness such that participants who recalled a positive memory tended to rate their memories as more meaningful than those who recalled a negative memory (*β* = .473, *t* = 9.722, *p* < .001). However, there was no main effect of positive self‐schema strength on meaningfulness (*β* = .041, *t* = .842, *p* = .401). Overall, the first step was significant, *F* (2, 330) = 47.668, *p* < .001, with memory valence and positive self‐schema strength accounting for 22.5% of the variance of participants' memory meaningfulness ratings. Contrary to our hypothesis, there was no significant interaction of memory valence and positive self‐schema strength on memory meaningfulness (*β* = .078, *t* = 1.157, *p* = .248).

##### Impact

**FIGURE 3:**
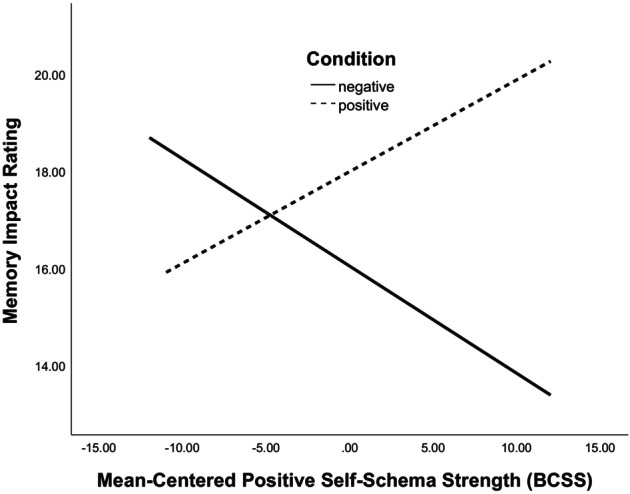
Interaction between positive self‐schema strength ratings (BCSS) and memory valence (assigned condition) on appraised memory impact. BCSS, Brief Core Schema Scale.

Results revealed a main effect of memory valence on impact, such that participants who recalled a positive memory tended to rate their memory as more impactful (*β* = 1.951, *t* = 2.537, *p* = .012). There was also a main effect of positive self‐schema strength on impact, such that participants with stronger positive self‐schemas tended to rate their memory as more impactful (*β* = −.221, *t* = −2.225, *p* = .027). Overall, the first step was significant, *F* (2, 332) = 3.039, *p* = .049, with memory valence and positive self‐schema strength accounting for 1.8% of the variance in participants' memory impact ratings. The second step was also significant, *F* (3, 332) = 4.824, *p* = .003, with the interaction between memory valence and positive self‐schema strength accounting for a total of 4.2% of the variance in participants' memory impact, Δ*R* ^2^ = .024. Consistent with hypotheses, there was a significant interaction between memory valence and positive self‐schema strength on impact (*β* = .410, *t* = 2.874, *p* = .004). Simple slopes analyses revealed that participants who endorsed stronger positive self‐schemas rated their negative memories as less impactful (*β* = −.177, *t* = −2.286, *p* = .024), but the impact of positive self‐schemas on positive memories only approached significance (*β* = .138, *t* = 1.800, *p* = .074), such that positive memories were rated as trending towards being more impactful by those with stronger positive self‐schemas (see Figure 3).

### Exploratory analyses

We also performed four sets of preregistered exploratory hierarchical linear regression analyses to examine the effects on memory appraisals of all three predictor variables and their interactions together in one model. Results indicated that SA was no longer a significant predictor of appraised memory impact when entered simultaneously in the model with negative self‐schema strength (see Table 4).

**TABLE 4:** Hierarchical multiple regression examining the influence of social anxiety symptoms, negative self‐schema strength and condition on memory impact ratings.

Outcome	Predictor	*β*	*t*	*R*	*R* ^2^	Δ*R* ^2^
Impact	Step 1					
	Social anxiety	.084	1.292	.237	.056***	.056
	Negative self‐schema	.133	2.039*			
	Condition	.134	2.450*			
	Step 2					
	Social anxiety	.103	1.049	.274	.075	.019
	Negative Self‐Schema	.240	2.176*			
	Condition	.138	2.508*			
	Social anxiety × Condition	−.049	−.547			
	Negative self‐schema × Condition	−.156	−1.743			
	Social Anxiety × Negative Self‐Schema	.005	.076			
	Step 3					
	Social Anxiety	.101	1.026	.278	.077	.003
	Negative Self‐Schema	.280	2.369*			
	Condition	.111	1.803			
	Social Anxiety × Condition	−.041	−.450			
	Negative Self‐Schema × Condition	−.216	−1.966			
	Social Anxiety × Negative Self‐Schema	−.050	−.542			
	Social Anxiety × Negative Self‐Schema × Condition	.085	.941			

*Note:* Social anxiety symptoms were measured via Social Phobia Inventory (SPIN) scores; negative self‐schema strength was measured via Brief Core Schema Scale (BCSS) negative self‐schema scores; condition was randomly assigned (positive vs. negative memory retrieval).

* *p <* .05.

*** *p* < .001.

## DISCUSSION

The purpose of this study was to examine the effects of positive versus negative social autobiographical memory retrieval, SA symptoms and positive and negative self‐schemas on appraised memory impact and meaningfulness. Results demonstrated that, overall, positive memories were rated as more impactful and meaningful than negative memories. This finding aligns with past research on positivity bias in the general population showing that positive social feedback is incorporated into self‐views to a greater extent than negative social feedback (Korn et al., 2012; Sharot et al., 2011; Taylor & Brown, 1988). The healthy bias contrasts with socially anxious individuals' tendency to magnify their perception of the influence of negative social experiences (e.g., Moscovitch et al., 2011, Moscovitch, Rowa, et al., 2015; Moscovitch, Waechter, et al., 2015; Moscovitch et al., 2018) and extract less positivity from social interactions (Clark & Wells, 1995; Glazier & Alden, 2019; Kashdan et al., 2011; Koban et al., 2017). Accordingly, in this study and in support of our hypotheses, individuals with higher SA (and more negative self‐schemas) tended to rate their negative social memories, but not their positive social memories, as more impactful on their views of themselves, others and the world. Conversely, the predicted interaction effects for appraised meaningfulness were not supported, though memory valence, social anxiety and self‐schemas each explained significant variance in meaningfulness as main effect predictors.

While, as expected, SA symptoms and negative self‐schemas each predicted elevated impact ratings of negative social memories, these factors did not predict lower impact ratings of positive social memories. The lack of an association between SA symptoms and reduced perceived impact of positive social memories contrasts with past research that has highlighted memory‐related positivity deficits in SA (Alden et al., 2008; Romano, Tran, & Moscovitch, 2020).

Considering both sets of findings together, this study suggests that positive social memories may be less influenced by SA than negative memories. One possible explanation for this is that positive social memories may undergo more deliberate and conscious processing and recollection due to their incongruence with active self‐schemas (Moscovitch, Moscovitch, & Sheldon, 2023), thus making their perceived impact less susceptible to the negativity bias induced by SA symptoms. Conversely, negative memories may be more automatically retrieved because the details and appraisals of such memories tend to align more closely and be more congruent with SA‐related self‐schemas, thereby increasing vulnerability to SA‐related processing biases. Nonetheless, it is possible that both negative and positive memories can be used therapeutically to drive schema‐incongruent learning.

To this end, recent studies have demonstrated the benefits of both imagery rescripting procedures of negative autobiographical memories (e.g., Romano, Ma, et al., 2020; Romano, Tran, et al., 2020) and intentional retrieval and deep processing of positive autobiographical memories (Moscovitch, White, & Hudd, 2023) for people with higher SA symptoms or SAD. Whereas imagery rescripting is designed to ‘unhook’ the self from a past negative experience that reinforces negative self‐schemas, positive memory self‐processing is designed to hook the self onto a positive past experience that reinforces positive self‐schemas. In this way, both types of memory‐based interventions are intended to induce schema change by activating the autobiographical memory system in accordance with the tenets of the Schema‐Congruent and ‐Incongruent Learning model (Moscovitch, Moscovitch, & Sheldon, 2023). According to the SCIL model, effective schema‐change interventions work through the mechanisms of repeated mental simulation and prediction error (Moscovitch, Moscovitch, & Sheldon, 2023). The SCIL model outlines a three‐stage process of schema updating that can be administered by clinicians via targeted interventions in which anxious patients are guided to recollect or imagine past or future experiences that activate the autobiographical memory system in ways that either weaken existing negative self‐schemas or strengthen new positive self‐schemas (Moscovitch, Moscovitch, & Sheldon, 2023; Moscovitch & Huppert, 2024).

Reflecting on the differing impacts of positive versus negative autobiographical memories on SA, it is noteworthy that individuals who struggle with SA tend to ruminate on negative, but not positive, social experiences (Clark & Wells, 1995). This ruminative process, called post‐event processing, involves repetitive self‐focused thoughts regarding feelings of anxiety, details from specific negative events and negative self‐perceptions (see Brozovich & Heimberg, 2008), during which SA‐related self‐schemas may influence memory encoding. In a seminal study on memory for feedback following a social task, researchers found that post‐event processing mediated the relationship between SA and negative memory bias for negative feedback items but did not mediate the relationship between SA and reduced positive memory bias for positive items (Cody & Teachman, 2010), suggesting that negative memories are more susceptible to post‐event processing, which could influence the future appraisals of a given memory by distorting the memory to seem more negative than it was originally appraised to be.

Beyond the effects of SA, results of this study also supported our hypothesis that participants who endorsed stronger negative views of themselves (e.g., I am unloved, worthless, weak, etc.) would rate their recollections of specific negative social experiences as more impactful. In contrast to our hypothesis, however, they did not rate their recollections of positive social experiences as less impactful, mirroring the findings on the impact of SA symptoms of negative and positive memories, discussed above. Overall, these findings are consistent with earlier studies showing that individuals with negative self‐schemas exhibit a preference for processing and storing schema‐consistent memories and have greater capacity to learn and retain social feedback that is consistent with such self‐schemas (e.g., Glazier & Alden, 2019). Notably, when SA symptoms and negative self‐schemas were entered into the same exploratory model, only negative self‐schemas remained a significant predictor, suggesting that it may be the negative self‐schemas, rather than SA symptoms *per se*, that impact and bias memory appraisals.

Due to the correlational nature of the study, it is not possible to conclude that social anxiety and self‐schemas exert a unidirectional or causal influence on autobiographical memory appraisal *per se*. Our study design does not rule out the possibility of a bidirectional relationship, such that people who are more socially anxious or have stronger negative self‐schemas tend to experience and encode more impactful negative events, which in turn then strengthen SA symptoms and negative self‐schemas. Indeed, self‐schemas are developed and strengthened through repeated but independent experiences that share common elements (Moscovitch, Moscovitch, & Sheldon, 2023). The negative impact of the recalled social memories in this study may have been compounded by other negative social experiences that worked together to strengthen participants' negative self‐schemas. Social anxiety is similarly impacted by negative social experiences. For example, people with SAD tend to recall negative social experiences using more detail and with more associated distress than people without SAD, which contributes to heightened negative self‐perceptions that underlie SA symptoms (Moscovitch et al., 2018; Reimer & Moscovitch, 2015).

In addition to our hypotheses concerning the effects of SA symptoms and negative self‐schemas on positive and negative memory appraisals, we further hypothesized that holding stronger positive self‐schemas (e.g., I am respected, valuable, successful, etc.) would be linked to recollecting more impactful and meaningful positive social experiences and less impactful and meaningful negative social memories. In partial support of this hypothesis, results showed that individuals with stronger positive self‐schemas tended to perceive their negative memories as less impactful. Additionally, though only approaching significance, we found that those with stronger positive self‐schemas rated their positive social memories as more impactful. These findings suggest that stronger positive self‐schemas could serve a protective function by reducing the impact of negative social autobiographical memories, though this hypothesized causal relationship requires verification in future experimental research.

### Limitations

The methodological limitations of our study must be acknowledged. First, the age of memories may have impacted participants' memory appraisals. More recent memories may be more vivid and therefore perceived as more impactful or meaningful, whereas memories that are older may have faded or been subject to more reconsolidation, potentially influencing their appraisal (Söderlund et al., 2014). Participants in this study recounted memories that spanned in age from under a week to over 15 years old (see Table 2), with approximately 40% of participants recollecting a memory that had occurred within the past 6–18 months. Future analyses could explore the potential relationship between memory age and ratings of memory impact and meaningfulness, and whether this relationship differs for positive versus negative memories, which could provide additional insight into how individuals interpret and make sense of their social experiences over time.

Importantly, our study was conducted on a non‐clinical community sample, so future replication and extension is needed on a clinical sample of participants who have a diagnosis of SAD.

An additional constraint with respect to our sample was the fact that a significant proportion of our respondents (77%) self‐identified as being White or Caucasian and residing in the United Kingdom (92%). Future research should aim to recruit a more culturally and geographically diverse sample of participants to examine the potential moderating influence of individual differences and enhance the generalizability of study findings.

Finally, the findings in this study were based almost exclusively on self‐report measures. Future studies could examine the linguistic properties of positive versus negative memory narratives (e.g., Moscovitch et al., 2011, 2018) to shed additional light on how participants' SA symptoms and self‐schemas are linked to memory encoding and retrieval processes. Future work should also incorporate behavioural outcome measures to investigate the effects of SA, self‐schemas and positive and negative autobiographical memory retrieval on *in vivo* responses to social reward and threat.

## CONCLUSION

Despite these limitations, this study contributes to the existing literature by demonstrating that individuals with higher SA symptoms or stronger negative self‐schemas tend to rate their negative memories as more impactful, whereas those with stronger positive self‐schemas tend to rate their negative memories as less impactful. These findings have important implications for the development of therapeutic interventions for SAD, as they highlight that positive and negative self‐schemas have the potential to influence the perceived impact and importance of positive and negative social experiences, opening opportunities to alter schemas in a manner that ultimately reduces SA symptoms.

## AUTHOR CONTRIBUTIONS

**Katie E. Martin:** Conceptualization; investigation; writing – original draft; methodology; visualization; formal analysis; data curation. **Sophie M. Kudryk:** Conceptualization; writing – review and editing; methodology; formal analysis; project administration; data curation; supervision. **David A. Moscovitch:** Conceptualization; supervision; writing – review and editing; resources; project administration; software; funding acquisition.

## CONFLICT OF INTEREST STATEMENT

This authors report no conflicts of interest.

## Supporting information


Data S1.

## Data Availability

The data that support the findings of this study are openly available in Open Science Framework at https://osf.io/g52pe.
