# Supplementary material for: Effects of social anxiety and self‐schemas on the impact and meaningfulness of positive versus negative social autobiographical memories
Source: Br J Clin Psychol. 2024 Sep 16;64(2):281–96. doi: 10.1111/bjc.12504 (PMC12057315; doi:10.1111/bjc.12504)
Supplement: Supplementary file 1 — Data S1. [file BJC-64-281-s001.docx]

Supplemental Materials for

**Effects of social anxiety and self-schemas on the impact and meaningfulness of positive versus negative social autobiographical memories**

**Method**

**Participants**

***Statistical Power***

A priori power analyses were conducted based on Brysbaert’s (2019) recommendations for minimum sample size requirements of 100 participants plus an additional 100 participants per predictor variable in planned regression analyses. We aimed to recruit a sample of 400 participants to enable sufficient power to detect a medium effect size with alpha = .05 given the preregistered hierarchical multiple regression analyses that included two main predictor variables (memory valence, SA symptoms or self-schema strength) and one two-way interaction variable (memory valence x SA symptoms OR memory valence x self-schema strength). We also conducted additional exploratory analyses examining a more complex model that included memory valence, SA symptoms, and self-schema strength as well as their interactions as predictor variables, which were preregistered as exploratory because we expected they may be underpowered.

**Measures**

***Memory Validity, Condition Adherence Check, and Attentiveness and Engagement Measures***

To ensure that participants successfully retrieved a true autobiographical memory, they completed a memory validity check immediately after memory retrieval by responding “yes” or “no” to the following prompt: “Is your memory of a specific event that occurred at a particular time and place in the past?” At the end of the study, participants filled out a five-item condition adherence check questionnaire to gauge the degree to which they followed the instructions to retrieve a negative or positive autobiographical memory that occurred at a specific time and place. Participants also filled out a three-item engagement check questionnaire to assess their attentiveness and engagement as they completed the study. Summaries of memory responses were also inspected by researchers to ensure participants adhered to study instructions.

*Adherence Check Items for Positive Condition*

1. To what extent were you able to recall a positive memory from your personal past?
2. To what extent were you able to recall the details of the positive memory from your personal past?
3. To what extent did you attempt to recall as many details as you could when sharing about your personal memory?
4. While re-living your memory, to what extent did you focus on memory details that were associated with feeling accepted, connected, or valued?

*Adherence Check Items for Negative Condition*

1. To what extent were you able to recall a negative memory from your personal past?
2. To what extent were you able to recall the details of the negative memory from your personal past?
3. To what extent did you attempt to recall as many details as you could when sharing about your personal memory?
4. While re-living your memory, to what extent did you focus on memory details that were associated with feeling rejected, lonely, or unvalued?

*Engagement Check Items*

1. I gave questions some thought before answering them.
2. I was distracted while reading or answering questions.
3. I answered questions honestly.

**Procedure**

***Memory Recall Prompts***

If a participant indicated that they could not retrieve an appropriate autobiographical memory, they received a prompt that was designed to help them consider contexts in which they may have experienced a positive or negative social situation. Participants received a maximum of three prompts, which were provided one at a time. In between each prompt, the participant was asked if they could recall an appropriate memory. In the negative condition, seven participants received one extra prompt and three people received two extra prompts. In the positive condition, one person received two extra prompts.

*Memory Prompts for Positive Condition*

1. “Think of a moment within your life when you experienced a specific social situation in which you felt connected, understood, or a sense of belonging. Are you able to bring such an experience to mind?”
2. “Think of a moment within your life when you experienced a specific social situation in which you felt supported, valued, or cared for. Are you able to bring such an experience to mind?”
3. “Think of a moment within your life when you experienced a specific social situation in which you felt comfortable, relaxed, or safe with them. Are you able to bring such an experience to mind?”

*Memory Prompts for Negative Condition*

1. “Think of a moment within your life when you experienced a specific social situation in which you felt embarrassed, uncomfortable, or ashamed. Are you able to bring such an experience to mind?”
2. “Think of a moment within your life when you experienced a specific social situation in which you felt lonely, isolated, or alienated. Are you able to bring such an experience to mind?”
3. “Think of a moment within your life when you experienced a specific social situation in which you felt rejected, unvalued, or misunderstood. Are you able to bring such an experience to mind?”

**Excluded Data**

Data were excluded from analyses for 57 participants, leaving a final sample of N = 343 for analyses. Participants were excluded from analyses for various reasons. First, eight participants were excluded from the analyses if their study completion time was more than 3.0 standard deviations slower than other participants (i.e., + 4270.41 seconds). Second, one participant was excluded for failing three out of the four condition adherence checks. Third, two participants were excluded for failing two of the three attention checks. Fourth, if participants failed one of the two validity check questions, their memory transcript was reviewed to determine if it was suitable for inclusion. Three participants were excluded on this basis because they selected a memory that was non-social in nature, or they selected a memory that was not a true autobiographical memory that happened at a specific time and place. The analysis of the engagement measure did not result in any exclusions, as all participants met the criteria by passing at least two out of the three items. Finally, 43 participants were disqualified from the study since they either failed to confirm their consent, did not provide a memory narrative, or encountered technical issues that prevented them from recording their memory narratives.

**Results**

**Exploratory Analyses: SA Symptoms and Self-Schemas as Predictors in the Same Model**

Four sets of exploratory analyses were conducted. Memory valence condition (positive versus negative), SA symptoms (SPIN scores), and BCSS self-schema scores were entered as predictor variables, with either impact or meaningfulness as the dependent variables. Two of the four analyses included BCSS negative self-schema scores and two included BCSS positive self-schema scores. Main effects were entered on step one, all two-way interaction terms were entered on step two, and the three-way interaction term was entered on step three. Full regression results are presented in Table S4 and Table 5. There were few notable significant effects and no significant three-way interaction effects. Importantly, in the model of memory impact that included memory valence condition, SA symptoms, and BCSS negative self-schema scores, only the strength of negative self-schemas (*β* = .133, *p* = .042), but not SA symptoms (*β* = .084, *p* = .197), exerted a significant effect on the perceived impact of memories, regardless of valence (see Table 5).

**Supplemental Tables**

**Table S1**

*Hierarchical multiple regression examining the influence of social anxiety symptoms and condition on memory outcomes*

| Outcome | Variable | β | *t* | *R* | *R^2^* | ΔR*^2^* |
| --- | --- | --- | --- | --- | --- | --- |
| Meaningfulness | Step 1  Social Anxiety  Condition | .054  .472 | 1.091  9.597*** | .480 | .231*** | .231 |
|  | Step 2  Social Anxiety  Condition  Social Anxiety  x Condition | .131  .474  -.114 | 1.966  9.656***  -1.717 | .488 | .238 | .007 |
| Impact | Step 1  Social Anxiety  Condition | .073  1.782 | 2.881**  2.281* | .209 | .044*** | .044 |
|  | Step 2  Social Anxiety  Condition  Social Anxiety x Condition | .122  1.816  -.105 | 3.535***  2.335*  -2.077* | .238 | .057* | .013 |

*Note:* Social anxiety symptoms were measured via Social Phobia Inventory (SPIN) scores, while condition was randomly assigned (positive vs. negative memory retrieval); **p < .05, **p < .01, ***p < .001.*

**Table S2**

*Hierarchical multiple regression examining the influence of negative self-schema strength and condition on memory outcomes*

| Outcome | Predictor | β | *t* | *R* | *R^2^* | | ΔR*^2^* |
| --- | --- | --- | --- | --- | --- | --- | --- |
| Meaningfulness | Step 1  Negative Self-Schema  Condition | .110  .474 | 2.269*  9.812*** | .485 | .236*** | .236 | |
|  | Step 2  Negative Self-Schema  Condition  Negative Self-Schema x Condition | .187  .474  -.114 | 2.841**  9.844***  -1.725 | .492 | .242 | .007 | |
| Impact | Step 1  Negative Self-Schema  Condition | .239  1.947 | 3.234**  2.538* | .220 | .048*** | .048 | |
|  | Step 2  Negative Self-Schema  Condition  Negative Self-Schema x Condition | .308  .138  -.195 | 4.207***  2.588*  -2.670** | .262 | .069** | .020 | |

*Note* Negative self-schema strength was measured via Brief Core Schema Scale (BCSS) negative self-schema scores, while condition was randomly assigned (positive vs. negative memory retrieval); **p < .05, **p < .01, ***p < .001*

**Table S3**

*Hierarchical multiple regression examining the influence of positive self-schema strength and condition on memory outcomes*

| Outcome | Predictor | β | *t* | *R* | *R^2^* | ΔR*^2^* |
| --- | --- | --- | --- | --- | --- | --- |
| Meaningfulness | Step 1  Positive Self-Schema  Condition | .008  6.229 | .134  9.843*** | .495 | .245*** | .245 |
|  | Step 2  Positive Self-Schema  Condition  Positive Self-Schema x Condition | -.054  6.266  .126 | -.645  9.888***  1.055 | .498 | .248*** | .003 |
| Impact | Step 1  Positive Self-Schema  Condition | -.014  2.513 | -.186  3.074** | .175 | .031** | .031 |
|  | Step 2  Positive Self-Schema  Condition  Positive Self-Schema x Condition | -.226  2.650  .405 | -2.048*  3.268**  2.657** | .230 | .053*** | .022 |

*Note:* Positive self-schema strength was measured via Brief Core Schema Scale (BCSS) positive self-schema scores, while condition was randomly assigned (positive vs. negative memory retrieval); **p < .05, **p < .01, ***p < .001*

**Table S4**

*Hierarchical multiple regression examining the influence of social anxiety symptoms, positive self-schema strength, and condition on memory impact ratings*

| Outcome | Predictor | | β | *t* | *R* | *R^2^* | ΔR*^2^* |
| --- | --- | --- | --- | --- | --- | --- | --- |
| Impact | Step 1  Social Anxiety  Positive Self-Schema  Condition | .165  .023 | | 2.863**  .409 | .211 | .044** | .044 |
|  | Step 2  Social Anxiety  Positive Self-Schema  Condition  Social Anxiety x Condition  Positive Self-Schema x Condition  Social Anxiety  x Positive Self-Schema | .221  -.079  .135  -.104  .157  -.040 | | 2.656**  -.941  2.453*  -1.297  1.935  -.717 | .263 | .069* | .025 |
|  | Step 3  Social Anxiety  Positive Self-Schema  Condition    Social Anxiety x Condition  Positive Self-Schema x Condition  Social Anxiety x  Positive Self-Schema  Social Anxiety x Positive Self-Schema x Condition | .221  -.079  .135  -.104  .157  -.041  .001 | | 2.620**  -.938  2.359*  -1.295  1.926  -.554  .012 | .263 | .069 | .000 |

*Note:* Social anxiety symptoms were measured via Social Phobia Inventory (SPIN) scores; positive self-schema strength was measured via Brief Core Schema Scale (BCSS) positive self-schema scores; condition was randomly assigned (positive vs. negative memory retrieval); **p < .05, **p < .01, ***p < .001*
